# Determination of Platycodin D and Platycodin D3 in Rat Plasma Using Liquid Chromatography-Tandem Mass Spectrometry

**DOI:** 10.1155/2014/231293

**Published:** 2014-01-30

**Authors:** Tae-Hyun Kim, Byung Eui Lee, Eun Joo Kim, Yong Seok Choi, Keun-Sung Lee, Hak Rim Kim, Hyung-Gun Kim

**Affiliations:** ^1^Bioresourres Regional Innovation Center, Soonchunhyang University, Asan 336-745, Republic of Korea; ^2^Industry-Academy Cooperation Foundation, Soonchunhyang University, Asan 336-745, Republic of Korea; ^3^Korea Institute of Toxicology, Korea Research Institute of Chemical Technology, Daejeon 305-343, Republic of Korea; ^4^College of Pharmacy, Dankook University, Cheonan 330-714, Republic of Korea; ^5^Department of Pharmacology, College of Medicine, Dankook University, Cheonan 330-714, Republic of Korea; ^6^Dankook Translational Research Center, Dankook University, Cheonan 330-714, Republic of Korea

## Abstract

*Platycodon grandiflorum* has long been used as a traditional oriental medicine for respiratory disorder. Platycodin D (PD) is known as the main component isolated from the root of PG. A simple and rapid liquid chromatography-tandem mass spectrometry (LC-MS/MS) method has been developed and validated for the quantitation of PD in rat plasma. Quantitation was performed on a triple quadrupole mass spectrometer employing electrospray ionization and multiple reaction monitoring in positive ion mode. The total chromatographic run time was 4.0 min, and the calibration curves of PD were linear over the concentration range of 50–10,000 ng/mL in rat plasma. The coefficient of variation and relative error at five QC levels were 1.0 to 8.8% and 0.7 to 8.7%, respectively. After a single oral administration of 500 mg/kg and a single intravenous administration of 25 mg/kg of 3% PD extract (a PG extract including 3% of PD), platycodin D and platycodin D3 were detected and pharmacokinetic parameters were estimated. The oral bioavailability of platycodin D and platycodin D3 was 0.29% and 1.35% in rats at 500 mg/kg of 3% PD extract of PG, respectively. The present method can be applied to pharmacokinetic analysis of platycodins and platycosides of the PG.

## 1. Introduction

Platycodi Radix, the root of *platycodon grandiflorum* (PG), is used extensively as an anti-inflammatory agent in the treatment of respiratory symptoms such as cough, sore throat, bronchitis, and bronchial asthma [1]. Some saponins such as platycodins (A, D, D2, and D3), polygalacin D2, platyconic acid A, and platycosides (A, B, C, D, E, and F) are separated from Platycodi Radix [2–5]. Platycosides are bidesmosidic saponins which have two sugar moieties, a glucose unit attached at C-3 of a triterpene and a series of three sugars (arabinose, rhamnose, and xylose in sequence) attached at C-28 through an ester linkage with the arabinose [2]. Platycosides have been known to have biological activities such as anti-inflammation, antiallergy, antitumor, immune response augmentation, antiobesity, and antihyperlipidemia [6–10]. Among them, platycodin D has been reported to inhibit COX-2 induction by 12-O-tetradecanoylphorbol-13-acetate (TPA) and to suppress the production of prostaglandin E2 in rat peritoneal macrophages [11, 12].

The determination of these bioactive platycosides has been carried out mainly by traditional high performance liquid chromatography (HPLC) using a UV detector [6, 13] or an evaporative light scattering detector (ELSD) [4, 14]. However, since saponins including platycosides have very unique properties such as very weak absorbance even at short wavelengths, high polarity, thermal lability, and very low volatility, their analyses based on traditional HPLC techniques are complicated [4].

Thus, LC coupled with mass spectrometry through electrospray ionization (LC-MS), which shows high sensitivity, rapid analysis time, and low levels of sample consumption, has become an alternative technique for structural analyses of saponins from crude extracts of herbal plants [15–17]. Recently, liquid chromatography and tandem mass spectrometry (LC-MS/MS), which can provide structural information as well as molecular weight information of individual components of a mixture, have been actively applied to analyses of platycosides [4, 18, 19]. However, as these studies also focused only on the structural analysis of saponins including platycosides from Platycodi Radix, an alternative method for the quantitative analysis of platycosides, which can be used for their pharmacological research and the quality assurance of Platycodi Radix, was still absent.

Therefore, we developed and validated a simple and rapid LC-MS/MS method for the quantitative analysis of platycodin D, a representative platycoside of Platycodi Radix in this study. Notoginsenoside R1 was employed as the internal standard and eluent from a hydrophilic interaction chromatography (HILIC) column was analyzed by multiple reaction monitoring (MRM, positive ion mode) in a mass spectrometer. The total chromatographic run time was 4.0 minutes and the analytical performance including linearity, accuracy, and reproducibility of the present method was good. Additionally, this method was successfully applied to simultaneous determination of platycodin D, other platycodins, and platycosides following intravenous or oral administration of *Platycodon grandiflorum* extract to rats. Therefore, this developed method can be applied for not only pharmacokinetic studies of platycodin D but also quantitative analysis of other platycodins and platycosides from herbs with minor parameter changes for various purposes.

## 2. Materials and Methods

### 2.1. Materials and Reagents

PD-3% (*Platycodon grandiflorum* extracts powder including 3% of platycodin D), platycodin D, platycodin D3, platycodin A, platycodin D2, platyconic acid A, and platycoside E standards were donated by B&C Biopharm (Suwon, Republic of Korea). Notoginsenoside R1 (internal standard) was purchased from Fleton Natural Products Co., Ltd. (St. Huaishu, Chengdu, China). Water was purified with a Milli-Q water purification system (Millipore, Bedford, MA, USA). All other chemicals and reagents were of analytical grade and used without further purification.

A stock solution of PD (5 mg/mL) was prepared in 50% methanol and dilutions of stock solution were made with 50% methanol. Standard solutions of PD in rat plasma were prepared by spiking with an appropriate volume (less than 1% of total plasma volume) of the diluted stock solution, giving final concentrations of 50, 100, 200, 500, 1000, 2000, 5000, and 10000 ng/mL. All the solutions were stored at 4°C and were brought to room temperature before use.

Quality control (QC) samples of PD were prepared at concentrations of 50, 150, 1000, 8000, and 10000 ng/mL by the same method used for standard solutions. The IS solution was also prepared in 50% methanol and diluted with 100% acetonitrile to give a final concentration of 250 ng/mL. Aliquots of spiked plasma samples were taken in Eppendorf tubes and stored at −70°C.

### 2.2. Animal Study

Male Sprague-Dawley rats with a body weight of 200–250 g were purchased from Daehan-Bio Link (Seoul, Korea). The rats were kept in the animal facility under the conditions of constant temperature of 23 ± 2°C, a relative humidity of 50 ± 10%, and illumination with 12 h light/dark cycles until the initiation of the experiment. All animals were fed with standard animal chow daily and had access to drinking water ad libitum. The protocol for this animal study was approved by the Review Committee of Animal Care and Use of Dankook University (Cheonan, South Korea). After 1-week acclimation, the carotid artery (for blood sampling) and the jugular vein (for drug administration) were cannulated with polyethylene tubes (Clay Adams, Parsippany, NJ, USA) under anesthesia using Zoletil 50 (10 mg/kg i.m.). Each cannula was exteriorized to the dorsal side of the neck. Each rat was housed individually in a rat metabolic cage (Daejong Scientific Company, Seoul, Korea) and allowed for 4-5 hrs to recover from anesthesia before the study began. Platycodin D was administered by intravenous infusion over 1 min via the jugular vein (25 mg/kg, 3% powder) or by oral gavage (500 mg/kg, 3% powder). The total injection volume was approximately 0.6 mL, *n* = 4, respectively. Blood samples were collected from the carotid artery at 0 (predose), 0.083, 0.167, 0.25, 0.5, 0.75, 1, 1.5, 2, 3, 4, 6, and 8 hr after intravenous dosing and at 0 (predose), 0.083, 0.167, 0.25, 0.5, 0.75, 1, 1.5, 2, 3, 4, 6, 8, and 20 hr after oral dosing. Approximately 120 *μ*L of blood were withdrawn at each time point, and a similar amount of heparinized saline was back-flushed. The blood was immediately centrifuged at 3,000 rpm for 10 min to obtain 50 *μ*L of plasma, and the plasma was stored at −70°C until analysis.

### 2.3. Plasma Sample Preparation

Acetonitrile (200 *μ*L) and notoginsenoside R1 (250 ng/mL) were added to a 50 *μ*L aliquot of thawed plasma. After vortexing for 30 sec, the mixture was centrifuged at 12,000 rpm and 4°C for 10 min. The supernatant (100 *μ*L) was transferred to clean 96-well plate. A 5 *μ*L aliquot of the solution was injected into the LC-MS/MS system.

### 2.4. Liquid Chromatography-Tandem Mass Spectrometry

The liquid chromatographic system was a Accela (Thermo Fisher Scientific Inc., Waltham, MA, USA) system equipped with a solvent delivery module and an autosampler (Nanospace SI-2 3133, Shiseido, Tokyo, Japan) and connected to a Discovery Max (Thermo Fisher Scientific Inc.) quadrupole tandem mass spectrometer equipped with electrospray ionization (ESI-MS-MS). System control and data analysis were carried out with Xcalibur (Thermo Fisher Scientific Inc.). Chromatographic separation was achieved using a HILIC HPLC column (Sepax Polar-Imidazole, 100 mm × 2.1 mm i.d., 3 *μ*m particle size, Sepax Technologies, Delaware, USA) protected by a guard column (Phenomenex C18, 4 mm × 2 mm, Phenomenex). A column oven (Nanospace SI-2 3004, Shiseido, Tokyo, Japan) was used online. The mobile phase of 0.1% formic acid (2 *μ*M sodium acetate)-water/acetonitrile (30 : 70, *v/v*) was run at a flow rate of 350 *μ*L/min.

The electrospray ionization (ESI) mass spectrometer was operated in the positive ion mode. Multiple reaction monitoring (MRM) of the precursor-product ion transitions from *m/z* 1247.7 to *m/z* 705.2 for PD, from *m/z* 1409.9 to *m/z* 867.5 for PD3, and from *m/z* 955.4 to *m/z* 775.4 for notoginsenoside R1 was used for quantitation. Collision energy was 62.0, 80.0, and 43.0 volts for PD, PD3, and notoginsenoside R1, respectively. The optimized conditions were ESI needle spray voltage (5000 V), sheath gas pressure (35 unit), auxiliary gas pressure (15 unit), capillary temperature (290°C), collision gas (Ar) pressure (2.0 mTorr), and skimmer offset (−2 V). The scan was performed in centroid mode with SIM width, 0.7 FWHM, scan time, 0.1 sec, and scan width 0.5 Da.

### 2.5. Method Validation

For the determination of linear range, 8 nonzero calibration samples were used. The linear regression of the ratio of peak area of PD divided by that of IS versus the concentration of PD was done with the weighting of 1/*X* ^2^ (least-squares linear regression analysis, where *X* is the analyte concentration). The lower limit of quantitation (LLOQ) was defined as the lowest concentration of PD with acceptable precision and accuracy (<±20%). For other concentrations, precision and accuracy should be within ±15%.

The precision and accuracy of this analysis method were determined by the concentration of the QC samples which were calculated from the simple linear equation at each day using regression analysis of spiked plasma calibration standard with reciprocate of the drug concentration as a weighting factor (1/concentration^2^, i.e., 1/*X* ^2^). Five replicates of QC samples were measured during a single run, and this was done on different five days. The accuracy and precision were evaluated in terms of relative error (RE) and % of CV, respectively.

The matrix effect of PD was determined by comparing the mean peak areas of the analyte spiked at three concentrations (150, 1000, and 8000 ng/mL) in the extracts originating from six different lots of rat blank plasma sample (set 1) to the mean peak areas for the neat solutions of the analyte in 75% acetonitrile (set 2). The recovery of PD was determined by comparing the mean peak areas of analytes spiked before extraction in the same six different sources as set 1 (set 3) with those of the analytes spiked after extraction into different blank plasma extracts at three concentrations, 150, 1000, and 8000 ng/mL (set 1). The matrix effect and recovery of IS were determined in one concentration (1000 ng/mL) using the same way for PD.

Stock solution stability was performed by comparing area response of stability sample of analyte and internal standard with the area response of sample prepared from fresh stock solutions. The stocks were found to be stable for a minimum of 2 weeks. The stability in extracted sample (postpreparative stability), the stability in rat plasma after three freeze/thaw cycles, the long term stability in rat plasma at –70°C for 2 weeks and 1 week, and the short-term stability in rat plasma at room temperature for 24 h (bench top stability) were measured by using five replicates of QC samples at three concentrations (150, 1000, and 8000 ng/mL), respectively.

## 3. Results

### 3.1. Mass Spectrometry

**Figure 1:**
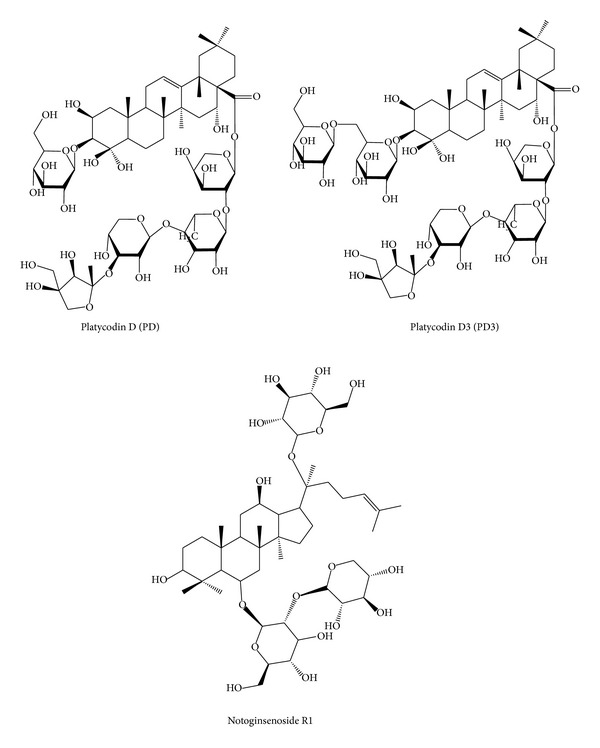
Chemical structures of platycodin D (PD), platycodin D3 (PD3), and notoginsenoside R1 (IS).

**Figure 2:**
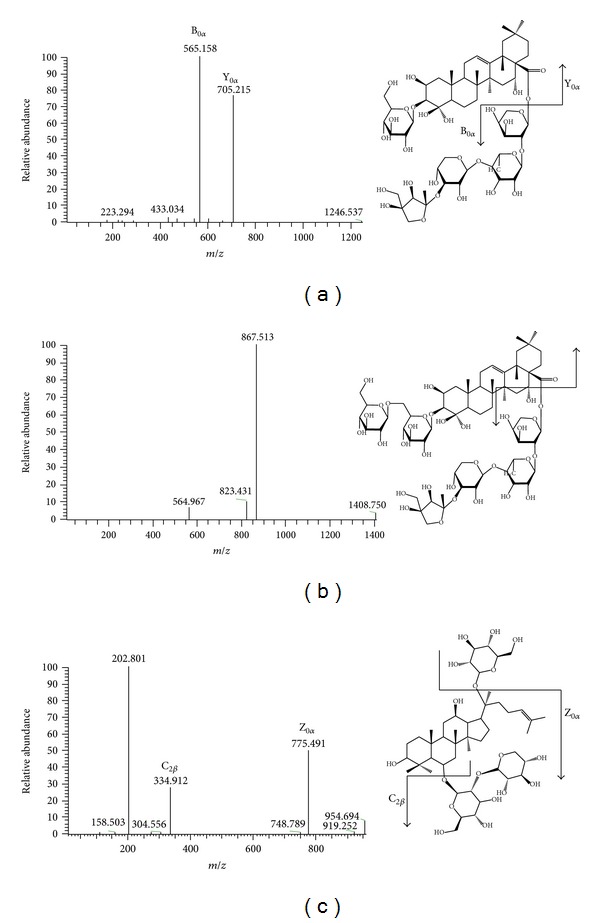
Product ion mass spectra used for building multiple reaction monitoring transitions for (a) PD (the precursor ion: [M+Na]^+^ at *m/z* 1247.7), (b) PD3 (the precursor ion: [M+Na]^+^ at *m/z* 1409.9), and (c) notoginsenoside R1 (the precursor ion: [M+Na]^+^ at *m/z* 995.4).

The chemical structures of PD, PD3, and IS are shown in Figure 1, and those were ionized under our electrospray conditions, and ionized analytes could be sensitively detected by a mass spectrometer in positive ion mode. The most abundant peak representing PD or PD3 in full mass spectra was its monosodiated ion peak ([M+Na]^+^, *m/z* 1247.7, and *m/z* 1409.9, resp.). The monosodium adduct peak of IS ([M+Na]^+^, *m/z* 955.4) was also its most abundant peak in full mass spectra. Product ions by ms/ms scans of the individual [M+Na]^+^ ions and their proposed fragmentation patterns (Y_0*α*_ ion (705.2 *m/z*) and B_0*α*_ ion (565.2 *m/z*) from PD) and (Y_0*α*_ ion (867.5 *m/z*) and B_0*α*_ ion (564.6 *m/z*) from PD3 and Z_0*α*_ ion (775.4 *m/z*) and C_2*β*_ ion (334.9 *m/z*) from IS) are shown in Figures 2(a), 2(b), and 2(c). As a result, MRM transitions for PD (*m/z* 1247.7 to *m/z* 705.2), PD3 (*m/z* 1409.9 to *m/z* 867.5), and IS (*m/z* 955.4 to *m/z* 775.4) were determined and various parameters for mass spectrometer were optimized by a direct infusion study of a PD standard solution.

### 3.2. Sensitivity and Specificity

**Figure 3:**
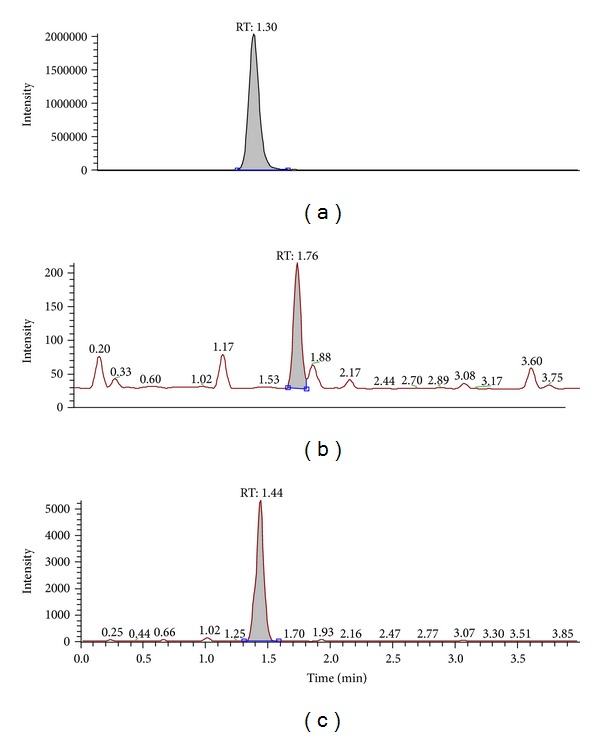
Representative chromatograms of rat plasma spiked with PD (50 ng/mL, LLOQ), PD3 (50 ng/mL, LLOQ), and IS ((a) PD, (b) PD3, and (c) IS).

**Figure 4:**
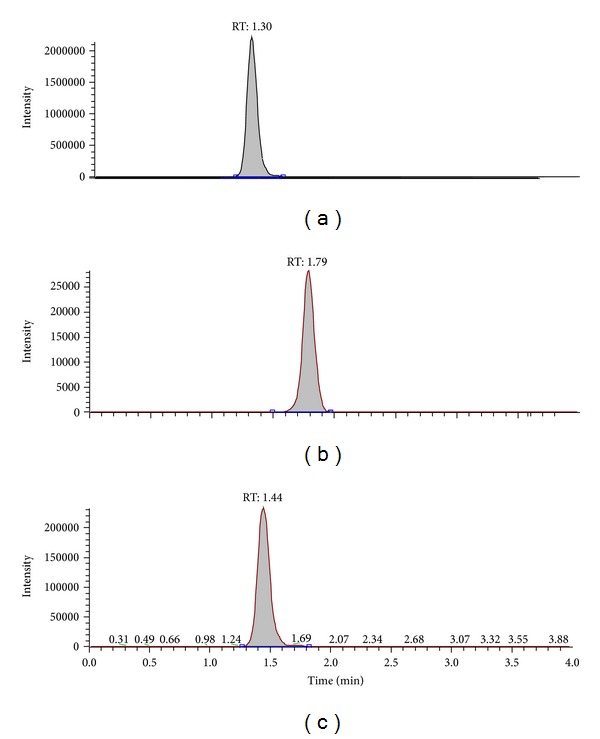
Representative chromatograms of plasma sample 60 min after intravenous administration of  25 mg/kg of PD-3% ((a) PD, (b) PD3, and (c) IS).

**Figure 5:**
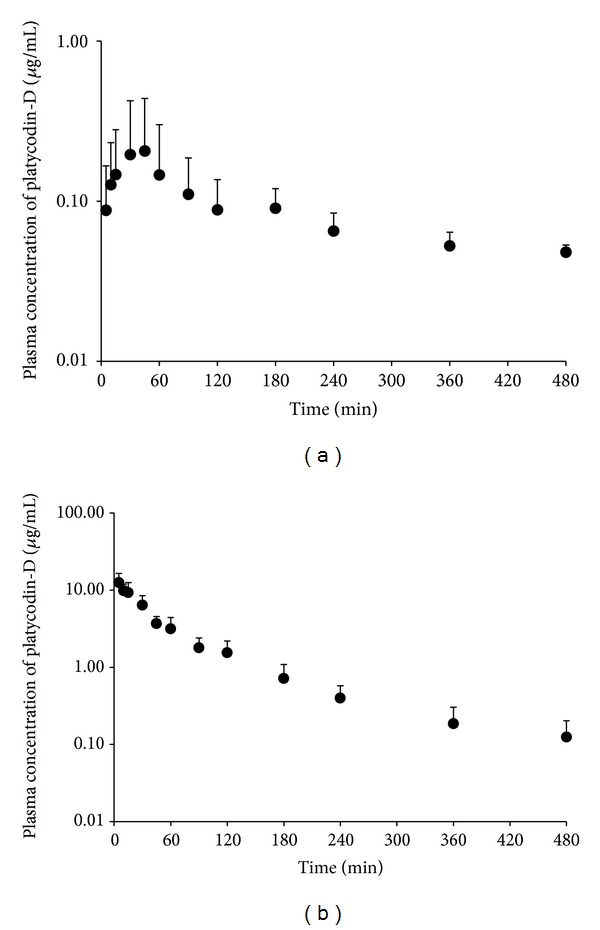
Mean plasma concentration-time profile of PD after intravenous administration of 25 mg/kg and oral administration of 500 mg/kg of PD-3% to rats (*n* = 4). Vertical bar represents standard deviation.

The representative chromatograms are from a drug-free rat plasma sample (Figure 3), a rat plasma validation sample at LLOQ with 50 ng/mL of PD and IS (Figure 4), and a plasma sample collected at 60 min after intravenous administration (Figure 5). No significant interference from endogenous substances was observed in 5 different sources of rat plasma samples. The limit of quantitation for PD in rat plasma was 50 ng/mL based on a signal-to-noise ratio of 10.0.

### 3.3. Linearity

Eight-point standard curves were linear over the concentration range of 50–10000 ng/mL for PD (Table 1). The correlation coefficients (*r* ^2^) of the standard curves generated during the validation ranged between 0.9976 and 0.9993. At all individual concentrations, RE values between nominal and measured concentrations ranged from −5.3 to 4.2%. CV values were limited to less than 6.0% through the study.

### 3.4. Precision and Accuracy

The intrabatch and interbatch precision and accuracy were evaluated at LLOQ, low QC, medium QC, high QC, and ULOQ concentrations. The ranges of intrabatch and interbatch precision CV of PD in rat plasma were 2.7–5.2% and 1.0–8.8%, respectively, and they were within the nominal concentration range of 50–10000 ng/mL (Table 2). The intrabatch and interbatch accuracies (RE) of PD were −1.3–3.1% and −8.7–3.5%, respectively, within the concentration range of 50–10,000 ng/mL (Table 2).

### 3.5. Recovery, Matrix Effect, and Stability

The results of the recovery, matrix effect of PD and IS are summarized in Table 3. In rat plasma, the matrix effects and recovery at three concentration of PD were 86.2–99.3% and 90.4–94.5%, respectively. It shows that the ionization suppression or enhancement was negligible in the analysis of PD in the present method. In the case of IS, the matrix effect at 1000 ng/mL was about 99.1%, but its recovery was 73.6%. Although the reasons of the relatively low IS recovery are unknown, it is still good for the quantification purpose because its value is higher than the acceptable criterion (at least 50%) for the biological sample recovery.

Stock solution of PD and IS was stable for 24 hours at room temperature and for 30 days at 4°C. After sample preparation, PD was stable for 24 hours in autosampler (4°C). In rat plasma, PD was stable after three freeze/thaw cycles. At room temperature, PD was also stable for at least 24 hours. Long term stability was also good at –70°C for 1-2 weeks. The results from stability tests of PD were summarized in Table 4.

### 3.6. Pharmacokinetics of PD

**Figure 6:**
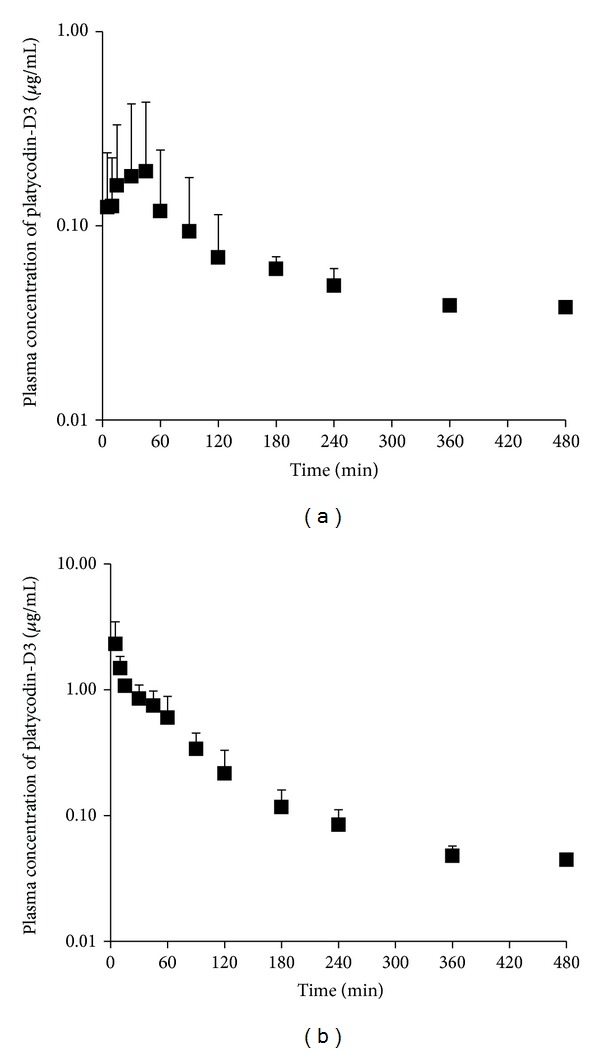
Mean plasma concentration-time profile of PD3 after intravenous administration of 25 mg/kg and oral administration of 500 mg/kg of PD-3% to rats (*n* = 4). Vertical bar represents standard deviation.

This LC-MS/MS method was successfully applied to a pharmacokinetic study in rats. The plasma concentration-time profile after a single intravenous and oral administration of PD-3% to four rats is shown in Figure 6, and relevant pharmacokinetic parameters are listed in Table 5.

In case of a single intravenous administration (25 mg/kg, PD-3%), the area under the plasma concentration-time curve (AUC_0–20 h_) was 11.42 ± 3.43 *μ*g·hr/mL, and the elimination half-life (T_1/2_) was 1.99 ± 0.41 h. In case of a single oral administration (500 mg/kg, PD-3%), the area under the plasma concentration-time curve (AUC_0–20 h_) was 0.66 ± 0.36 *μ*g·hr/mL and the elimination half-life (T_1/2_) was 6.23 ± 1.84 hr. The bioavailability of PD (2,000 mg/kg, PD-3%) is 0.29% in SD rat.

This method could measure other platycodins and platycosides standards in the rat plasma, but only PD and PD3 were detected in the plasma in this study.

In case of a single intravenous administration (25 mg/kg, PD-3%), the area under the plasma concentration-time curve (AUC_0–20 h_) of PD3 was 1.96 ± 0.50 *μ*g·hr/mL, and the elimination half-life (T_1/2_) was 3.55 ± 0.87 h. In case of a single oral administration (500 mg/kg, PD-3%), the area under the plasma concentration-time curve (AUC_0–20 h_) of PD3 was 0.53 ± 0.31 *μ*g·hr/mL, and the elimination half-life (T_1/2_) was 6.20 ± 1.05 hr. The bioavailability of PD3 (2,000 mg/kg, PD-3%) is 1.35% in SD rat.

## 4. Discussion

In this study, we developed a simple and rapid LC-MS/MS method for the quantitation of PD in rat plasma and also, we validate this newly developed method for PD analysis in plasma. We used acetonitrile for protein precipitation and notoginsenoside R1 was employed as an internal standard in the method for better performance. We found that the optimized and validated new method determining the terminal-phase levels of PD is appropriate for the purpose of pharmacokinetic study. We further studied the pharmacokinetic profile of PD and PD3 after administration of PD-3% (*Platycodon grandiflorum* extracts powder including 3% of platycodin D). To the best of our knowledge, this is the first pharmacokinetic report of simultaneous determination of PD and PD3 following intravenous or oral administration of PG extract to rats. In this study, HILIC column was used for simple isocratic separation with short running time (4 min). Therefore, this method could be very useful not only for additional pharmacokinetic studies of PD but also for the development of MRM methods targeting other platycosides.

Previously, Pei et al. reported that the bioavailability was 0.079% after intravenous and oral administration of pure PD (>98%) [20]. The bioavailability of PD and PD3 is 0.29% and 1.35%, respectively, in our study. The differences between two studies suggest that the bioavailability of PD could be changed by the extraction method of PG or by coadministered or coextracted compounds in the PG extracts. The relatively big difference of oral bioavailability between PD and PD3 and related factors for enhancing gastrointestinal absorption of PD should be further evaluated by applying present method to estimate the pharmacokinetic parameters of PD following intravenous or oral administration of various PG extract to rats.

## Figures and Tables

**Table 1:** Calculated concentrations of PD in calibration standards prepared in rat plasma (*n* = 5 at each level).

	Theoretical concentration (ng/mL)								Slope	Intercept	*r* ^2^
	50	100	200	500	1000	2000	5000	10000			
Mean	50.9	98.7	191.1	493.5	1000.2	2031.5	5027.2	10309.1	0.00008	−0.001905	0.9982
CV (%)	3.2	6.1	5.1	5.1	4.5	6.3	3.0	2.7			
RE (%)	1.8	−1.3	−4.5	−1.3	0.0	1.6	0.5	3.1			

CV: coefficient of variation; RE: relative error.

**Table 2:** Intrabatch and interbatch precision and accuracy of PD in rat plasma.

Nominal concentration (ng/mL)	Measured concentration (ng/mL)	CV (%)	RE (%)
Intrabatch^a^			
50	50.9 ± 1.6	3.2	1.8
150	148.0 ± 5.5	3.7	−1.3
1000	1007.3 ± 44.2	4.4	0.7
8000	7913.0 ± 411.6	5.2	−1.1
10000	10309.1 ± 282.0	2.7	3.1
Interbatch^b^			
50	50.4 ± 2.0	4.0	0.7
150	136.9 ± 7.0	6.2	−8.7
1000	968.3 ± 75.3	8.0	−3.2
8000	7877.1 ± 693.6	8.8	−1.5
10000	10101.7 ± 352.4	1.0	3.5

CV: coefficient of variation; RE: relative error.

^a^Comparisons of five replicates (*n* = 5) observations at each concentration.

^b^Comparisons of 25 replicates (*n* = 5) observations over five different analytical runs.

**Table 3:** Recovery and matrix effect of platycodin D and IS in rat plasma (*n* = 4 at each level).

Nominal concentration	Absolute matrix effect (%)	Recovery (%)
Platycodin D		
150 ng/mL	86.2	94.5
1000 ng/mL	90.4	92.0
8000 ng/mL	99.3	90.4
Notoginsenoside R1 (IS)		
1000 ng/mL	99.1	73.6

Absolute matrix effect was expressed as the ratio of the mean peak area of an analyte spiked into plasma extract after acetonitrile treatment to the mean peak area of the same analyte standards in mobile phase multiplied by 100.

Recovery was calculated as the ratio of the mean peak area of an analyte spiked into plasma before acetonitrile treatment to the mean peak area of an analyte spiked into plasma extract after acetonitrile treatment multiplied by 100.

**Table 4:** Stability of PD in rat plasma (*n* = 5 at each level).

Conditions	Theoretical concentration (ng/mL)	Final concentration (ng/mL)	RE (%)	CV (%)
Three freeze/thaw cycles (−70°C)	150	149.8	0.1	4.2
	1000	1005.3	0.5	3.7
	8000	7956.8	−0.5	3.2

Bench top stability (room temp., 24 h)	150	147.9	−1.4	7.0
	1000	1053.5	5.4	5.2
	8000	8180.9	2.3	1.8

Postpreparative stability (4°C, 24 h)	150	147.0	−2.0	6.2
	1000	927.1	−7.3	1.1
	8000	7479.3	−6.5	1.3

Long term stability (−70°C, 1 week)	150	148.4	−1.1	5.5
	1000	973.9	−2.6	3.1
	8000	7425.3	−7.2	1.9

Long term stability (−70°C, 2 weeks)	150	152.2	1.5	3.7
	1000	993.3	−0.7	6.1
	8000	7477.6	−6.5	3.6

CV: coefficient of variation; RE: relative error.

**Table 5:** Pharmacokinetic parameters of PD and PD3 after intravenous (25 mg/kg) or oral administration of PD (500 mg/kg) to rats (*n* = 4).

Parameter	PD	PD3
Intravenous		
AUC (*μ*g·hr mL^−1^)	11.42 ± 3.43	1.96 ± 0.50
Terminal half-life (hr)	1.99 ± 0.41	3.55 ± 0.87
MRT (hr)	1.58 ± 0.41	2.85 ± 0.77
*V*_ss_ (L·kg^−1^)	3.59 ± 1.28	35.32 ± 18.69
CL (L·hr^−1^ kg^−1^)	2.32 ± 0.90	11.84 ± 27.77
Oral		
AUC (*μ*g·hr mL^−1^)	0.66 ± 0.36	0.53 ± 0.31
Terminal half-life (hr)	6.23 ± 1.84	6.20 ± 1.05
*C*_max⁡_ (*μ*g·mL^−1^)	0.22 ± 0.22	0.20 ± 0.24
*T*_max⁡_ (hr)^a^	0.75 (0.17–3)	0.48 (0.11–1.61)

Data are mean ± SD (standard deviation).

^a^Median (ranges).
